# Effect of Ultrasound Pretreatment Combined with Magnetic Field-Assisted Freezing on Bioactive Compounds and Antioxidant Capacity of Blueberry

**DOI:** 10.3390/foods14234013

**Published:** 2025-11-23

**Authors:** Kaiyan You, Xuefeng Xiong, Ziyi Wang, Qianyu Li, Xuehui Cao

**Affiliations:** College of Food Science and Technology, Bohai University, Jinzhou 121013, China

**Keywords:** blueberry, magnetic field, bioactivity, antioxidant capacity, color

## Abstract

Magnetic field-assisted freezing is a novel freezing technology for improving food quality. The purpose of this study was to evaluate the effect of ultrasound pretreatment in combination with a static magnetic field on frozen blueberry bioactive compounds and antioxidant capacity. Blueberries were subjected to ultrasound pretreatment and frozen by using a static magnetic field. Bioactive components, antioxidant properties, organic acids, and the flesh color of blueberry were analyzed. The results demonstrated that, compared with -20 °C control freezing (CF), the ultrasound pretreatment combined with static magnetic field freezing (U-MF) better maintained blueberry bioactive components. The anthocyanin and total phenolic and flavonoid contents of U-MF were 33.67%, 29.14%, and 18.65%, respectively, higher than the CF group (*p* < 0.05). The DPPH and ABTS free radical scavenging capacities of U-MF were 53.87% and 15.14%, respectively, higher than those of the CF group (*p* < 0.05). This article provides a theoretical basis for the application of magnetic fields in the frozen preservation of fruits and vegetables.

## 1. Introduction

Blueberries (*Vaccinium* spp.) contain a variety of vital nutrients (vitamins, minerals, and different antioxidants) which provide a number of health benefits [1]. There are different bioactive substances in blueberries, such as anthocyanins, flavonols, phenols, and proanthocyanidins [2]. However, blueberries are more susceptible to mechanical damage, which damages the tissue texture and leads to moisture and nutrient loss, thereby substantially reducing their shelf life [3]. Freezing is currently one of the most appropriate methods for the retention of the nutritional value of raw fruits and vegetables. Ice crystal formation can lead to irreversible damage to the tissue organization of fruits such as blueberries due to their high water content.

As a novel and safe freezing method, magnetic field-assisted freezing has gained extensive attention from researchers. In recent years, relevant studies have indicated that magnetic field-assisted freezing has been utilized extensively in fruits and vegetables [4,5]. Tang [6] demonstrated that a magnetic field not only reduces the volume of ice crystals and shortens the phase transition time, but also is a non-contact technology. A static magnetic field can alter the hydrogen bonding interactions within water molecules, causing them to align in an orderly fashion. This reduces the nucleation energy barrier, thereby promoting the formation of small, uniform ice crystals and minimizing the damage to cellular tissue. Ultrasound is an efficient, convenient, and safe non-thermal technology which can reduce enzyme activities (polyphenol oxidase, peroxidase, and lipoxygenase), as well as enhance the preservation of polyphenols, anthocyanins, and carotenoids [7,8]. Ultrasound may cause negative impacts on bioactive compounds, but because its non-thermal effect (50 kHz and 200 W) avoids thermal degradation, it promotes the release of bound phenolics.

Consequently, the aim of this paper was to investigate the influence of ultrasound pretreatment and static magnetic field freezing on the physicochemical characteristics of blueberries. The antioxidant capacity of blueberries was assessed using DPPH and ABTS. The organic acids and flesh color were used to examine the nutrition, brown stain of the fresh, and treated blueberries. The findings of this study provide insights into the effect of ultrasonic pretreatment combined with static magnetic field-assisted freezing on blueberry preservation.

## 2. Materials and Methods

### 2.1. Materials

Blueberries (variety “Hokuriku”) were harvested at Longshuo Blueberry Orchard (Jinzhou, Liaoning, China). The average mass and diameter of 25 kg of blueberries were 1.50 ± 0.10 g and 8–12 mm, respectively. Individual berries with similar size, shape, and maturity were carefully identified and immediately sent to the laboratory. To maintain a consistent initial temperature before the freezing treatment, blueberry samples were stored in a 4 °C refrigerator for 12 h.

### 2.2. Freezing Treatment

All fresh blueberries were divided into five groups (5 kg of blueberries in each group). The 5 kg of blueberries were separately placed in five trays (external dimensions: 23.5 × 15 × 5.8 cm). Five groups of blueberries were assigned to five different treatments: fresh blueberry (CK), control freezing (CF, 0 mT), static magnetic field freezing (MF, 10 mT), ultrasound pretreatment-assisted control freezing (U-CF, 0 mT), and static magnetic field freezing with the aid of ultrasonic pretreatment (U-MF, 10 mT). All blueberry samples of U-CF and U-MF were immersed in osmotic dehydration solution (1% calcium chloride and 5% trehalose) at a ratio of 1:5 (blueberries/solution) and placed in an ultrasonic bath (KQ-500DE, Kunshan Ultrasonic Instrument Co., Ltd., Kunshan, China; internal dimensions: 500 × 300 × 150 mm) working continuously at a frequency of 50 kHz for 5 min, with the power being set to 200 W. The static magnetic field-assisted freezing system contained two neodymium–iron–boron permanent magnets (100 mm × 50 mm × 10 mm) that were placed on both sides of the sample tray (23.5 × 15 × 5.8 cm) to generate a 10 mT static magnetic field (measured by a Gauss meter HT201 at the tray center). The ultrasound bath (KQ-500DE) operated at 50 kHz and 200 W in continuous mode. Subsequently, all freezing groups were marked and directly frozen in a −20 °C MFI-F1 refrigerator (INDUC Scientific Co., Ltd., Wuxi, China).

All freezing samples were individually freeze-stored in marked sealed bags in a −18 ± 1 °C refrigerator (BCD-408WBPBU1 Haier refrigerator, Haier Electric Co., Qingdao, China) for 72 h after being frozen. The research shows that a 72 h freezing time can ensure that the food is completely frozen and the internal ice crystal structure becomes more stable, resulting in less impact on the outcome. Frozen samples were thawed at 4 °C for 2 h in a refrigerator (BCD-408WBPBU1). The indicators were measured.

### 2.3. Anthocyanin Content

The anthocyanin content was determined using the spectrophotometric pH-differential technique [9]. Thawed blueberry samples (5.0 g) were homogenized in 60% ethanol solution (the acidity was measured in terms of acetic acid, and its value was 1.2 mg/L). The mixture was kept at 40 °C for 2 h and centrifuged for 20 min (4 °C) at 12,000× *g*. The supernatant (1 mL) was separated with 9 mL potassium chloride buffer (0.025 M and pH 1.0) and sodium acetate buffer (0.4 M and pH 4.5), respectively. Absorbances at 510 nm and 700 nm were assayed using a L9 Plus model UV–visible spectrophotometer (INESA Analytical Instrument Co., Ltd., Shanghai, China). The anthocyanin content of blueberries was calculated according to the following equation:

(1) $$\mathrm{Anthocyanin}\;\mathrm{content}\;(\mathrm{mg}/g)\;=\;\frac{OD\;\times \;MW\;\times \;f\;\times \;1000}{29600}$$

where OD (absorbance) = (OD_510 nm_ − OD_700 nm_) pH 1.0 − (OD_510 nm_ − OD_700 nm_) pH 4.5, MW (molecular weight) = 449 g mol^−1^ for cyanidin-3-glucoside (cyd-3-glu) (mL), and f is the dilution factor, where 29,600 represents the molar absorptivity (L·mol^−1^·cm^−1^) of cyanidin-3-glucoside (the main blueberry anthocyanin) at 510 nm.

### 2.4. Total Phenolic and Flavonoid Contents

Thawed blueberry samples (1.0 g) were homogenized in 80% methanol solution (25 mL), mixed and left in a 30 °C water bath for 1 h, and then centrifuged for 30 min at 8000× *g* (4 °C). The mixture was extracted twice and the supernatant was combined. The supernatant was volume-fixed at 100 mL using methanol (80%). Then, the extract was stored at 4 °C for use.

The total phenolic content of blueberries was assayed using the Folin–Ciocalteu colorimetric method [10] with some modifications. An amount of 0.8 mL of the extract was added to 10-fold diluted Folin phenol reagent (4 mL) and Na_2_CO_3_ solution (6 mL), with a mass fraction of 10%, avoiding light for 2 h at 25 °C. The standard sample was gallic acid. Absorbance at 765 nm was also measured.

The flavonoid content of blueberries was assayed using the NaNO_2_-Al (NO_3_)_3_-NaOH method [11]. The extract (2 mL) was added to the 0.5 mL NaNO_2_ solution (5%) and reacted for 6 min. Thereafter, 10% Al (NO_3_)_3_ solution (0.5 mL) was added and reacted for 6 min. A 4% NaOH solution (5 mL) was added, reacted for 15 min, and fixed to 10 mL of sample with distilled water. The standard sample was rutin. Absorbance at 510 nm was also measured.

### 2.5. Total Soluble Solid, Titratable Acidity, Ascorbic Acid, and Reducing Sugar Contents

The soluble solid (TSS), titratable acidity (TA), ascorbic acid, and reducing sugar contents were measured using the approaches proposed by Ullrich and Zhang [12,13,14]. TSS content was determined using a PAL-3 saccharimeter (Atago pocket PAL-3, Atago Co., Tokyo, Japan). TA content was determined using 0.1 mol L-1 sodium hydroxide solution. Ascorbic acid content was determined by potassium iodate titration. The determination of the reducing sugar content was performed with 3,5-dinitrosalicylic acid (DNS) using the colorimetric method [12]. Briefly, 1.0 g of blueberry samples was homogenized using 3.0 mL of distilled water, fixed to 25 mL of distilled water. The mixture was continually maintained at 80 °C for 30 min and then centrifuged at 12,000× *g* for 30 min (4 °C). The mixture was extracted twice. Subsequently, the collected supernatant was diluted and the volume was adjusted to 100 mL as an extraction solution. Afterward, 2 mL of the extraction solution was mixed with DNS (1.5 mL), followed by boiling for 5 min. Distilled water was added to adjust the volume to 25.0 mL; absorbance at 540 nm was then measured.

### 2.6. Organic Acid Content

The organic acid content of blueberries was assayed using high-performance liquid chromatography (HPLC) [15]. Thawed blueberry samples (1.5 g) were homogenized in ultrapure water (3 mL). The mixture was continually incubated for 30 min at 50 °C and centrifuged for 20 min at 8000× *g* (4 °C). Then, 0.45 μm syringes were used to filter the supernatant, which was packed into vials and stored at 4 °C for analysis.

A ZORBAX Eclipse Plus C 18 column (particle size 5 μm, 4.6 × 250 mm) was used for the HPLC (Agilent-1260, Agilent Technologies, Inc., Santa Clara, CA, USA) analysis. Methanol and 0.01 mM potassium dihydrogen phosphate (pH 2.5) at a ratio of 3:97 was used as the mobile phase, utilizing the isocratic system. The mobile phase flow rate was 0.6 mL min-1 and the column temperature was 30 °C. The injection volume was 10 μL, whereas the detection wavelength was 210 nm. Standard curves used four organic acid standard solutions of HPLC grade, including citric, malic, succinic, and quininic acids. These organic acid standards were purchased from Solarbao Science and Technology Co., Ltd. (Beijing, China).

### 2.7. Antioxidant Capacities

Thawed blueberry samples (1.0 g) were homogenized in acidified ethanol solution (10 mL) and extracted for 30 min in an ultrasound bath, and then centrifuged for 20 min at 10,000× *g* (4 °C), which was repeated three times. Afterward, all sample extracts were collected at 4 °C to analyze antioxidant capacities.

The radical scavenging activity of DPPH was assessed using 1,1-diphenyl-2-picrylhydrazyl [16], with slight modifications from the original method. The extract (2 mL) was combined with 2 mL of 0.1 mM DPPH solution in ethanol and stored in the dark for 30 min at 25 °C. Absorbance A1 at 517 nm was measured. The extract (2 mL) was mixed with ethanol (2 mL), and absorbance A2 at 517 nm was measured. The solution (2 mL) of 0.1 mM DPPH in ethanol was added to ethanol (2 mL), and absorbance A0 at 517 nm was measured.

(2) DPPH radical scavenging activity = (1 − (A_1_ − A_2_)/A_0_) × 100%

ABTS radical scavenging activity was determined using a previous method [17] with slight modifications. The sample extract (1.0/mL) was added to ABTS solution (3.0 mL). A solution: 0.192 g ABTS was accurately weighed and volume filled to 50 mL with distilled water; B solution: 0.067 g potassium persulfate was accurately weighed and volume filled to 50 mL with distilled water. A and B were mixed in equal volumes and stored in darkness for 12 h to produce the ABTS solution, which was diluted using ethanol. The absorbance (0.70 ± 0.02) at 734 nm was then obtained. The sample mixture was mixed and stored for 5 min in a 30 °C water bath; then, absorbance A1 at 734 nm was measured. A control sample (1 mL of distilled water and 3 mL of ABTS solution) was also prepared in parallel and absorbance A2 was measured.

(3) ABTS radical scavenging activity = (1 − A_1_/A_2_) × 100%

### 2.8. Color

The colors (L*, a*, and b*) of fresh and thawed blueberry flesh were analyzed using a spectrophotometer CR-400 (Minolta Japan). Blueberry flesh color data were expressed using the CIE L*a*b* scale: L* (brightness), a* (redness), and b* (blueness). The ΔE (total color difference) was calculated using fresh blueberry (CK) as the reference, with the following formula [18]:

(4) ΔE = √[(L* − L_0_*)^2^ + (a* − a_0_*)^2^ + (b* − b_0_*)^2^]

where L_0_*, a_0_*, and b_0_* are the color parameters of a standard plate (L_0_* = 94.60, a_0_* = −0.80, b_0_* = 3.91).

### 2.9. Statistical Analysis

All blueberry samples were measured in triplicate. The results are shown as means ± standard deviations. Data were processed using Excel 2016 software. A significance analysis was performed using IBM SPSS 27.0, with significance determined at *p* < 0.05. Mean comparisons were generated using the Duncan test. The figures were created using Origin 2021 software.

## 3. Results and Discussion

### 3.1. Anthocyanin Content

**Figure 1:**
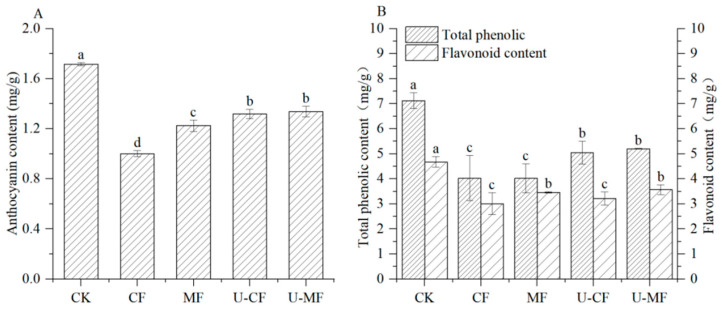
Anthocyanin (**A**) and total phenolic and flavonoid (**B**) contents of blueberries in different treatment groups. CK: fresh blueberries; CF: control freezing (−20 °C, 0 mT); MF: static magnetic field freezing (−20 °C, 10 mT); U-CF: ultrasound pretreatment + control freezing; U-MF: ultrasound pretreatment + static magnetic field freezing. All frozen groups were stored at −18 ± 1 °C for 72 h before analysis. Different letters indicate significant differences (*p* < 0.05).

Anthocyanins are a family of polyphenols which make up 60% of all polyphenols found in mature blueberries [19]. As a result, anthocyanins account for the majority of the blueberry’s health advantages. As shown in Figure 1A, the anthocyanin content of frozen blueberries exhibited a declining trend compared with fresh blueberries. The loss of anthocyanin content could be attributed to enzyme-catalyzed and oxidative reactions after the freezing of the berries, as well as a leakage of anthocyanins due to the diffusion and loss of water after thawing [20]. This causes ice crystals to melt, forming cavities within cells and facilitating the leakage of water-soluble compounds—such as anthocyanins and ascorbic acid—through damaged cell membranes. The anthocyanin content of CF was less than that of MF, U-CF, and U-MF after freezing and thawing. This implied that maintaining the anthocyanin content was positively impacted by the static magnetic fields and ultrasonic therapy. There was no significant difference in the anthocyanin content between U-MF and U-CF (*p* > 0.05). U-CF maintained a higher content of anthocyanins in the frozen blueberries when compared to CF. Ca^2+^ in the osmotic solution cross-links with pectin in the cell walls to stabilize the membrane structure, reducing water and nutrient loss while maintaining texture and preventing tissue softening caused by freezing. Furthermore, trehalose, acting as an antifreeze agent, inhibits ice crystal growth. This could be due to the fact that ultrasound processing contributes to the disruption of the cellular plant tissue, facilitating the release of previously bound phenolics [20]. A previous study showed that ultrasound treatment can release intermediates and increase the bioactive compound content [8].

### 3.2. Total Phenolic and Flavonoid Contents

Figure 1B depicts the change in the total phenolic and flavonoid contents of blueberries. The total phenolic content of all treatment blueberries demonstrated a significant declining trend compared with the fresh blueberries. No significant differences were observed in the overall phenolic contents of blueberries treated with CF and MF (*p* > 0.05), or those treated with U-CF and U-MF, indicating that static magnetic field freezing had no obvious effect on the total phenolic content of the blueberries. However, ultrasound processing enhanced the overall phenolic content of the samples. A study was carried out using ultrasound (180 W power) to treat apple tissues, and the total phenol content was obviously increased in all of the groups treated compared to that of the conventional refrigerated group [7]. As shown in Figure 1B, the flavonoid content of blueberries decreased after freezing and thawing. No significant differences were observed in the total flavonoid contents of blueberries treated with CF and U-CF (*p* > 0.05), or those of blueberries treated with MF and the U-MF (*p* > 0.05). These findings suggest that ultrasound had no significant influence on the flavonoid content of blueberries. Previous studies concluded that there was no modification to the flavonoid contents of blueberry and cranberry samples after ultrasound treatment compared with the control sample [21,22]. However, the blueberries treated with MF and U-MF maintained a high flavonoid content compared with CF (Figure 1B). Ref. [23] showed that a magnetic field could delay the decline in the flavonoid content of strawberry, which was related to the conversion of flavonoids to other secondary phenolic compounds during fruit ripening and senescence.

### 3.3. TSS, TA, Ascorbic Acid, and Reducing Sugar Contents

TSSs and reducing sugars supply the carbon skeletons and energy needed by cells [24]. TA is an important parameter impacting fruit and vegetable flavor quality [25]. Ascorbic acid is a crucial reducing substance of the ascorbate–glutathione cycle, which can effectively remove reactive oxygen species and increase the antioxidant ability of fruit [23]. TSS, TA, ascorbic acid, and reducing sugar contents are summarized in Table 1. The TSS and decreasing sugar contents of all treatment blueberries showed a significant increasing trend compared with the fresh blueberries (*p* < 0.05). TA and ascorbic acid levels in all treatment blueberries decreased significantly as compared to fresh blueberries (*p* < 0.05).

Among them, compared with CF, there was a substantial difference in the TSSs of MF, and U-MF, but no significant difference in U-CF, which indicated that the static magnetic field had a significant effect on TSSs. It can be seen from the Table 1 that the TA was significantly lowered by 32.26% in MF, 48.39% in U-CF, and 67.74% in U-MF compared to CF. The TA of MF, U-CF, and U-MF was significantly different compared with CF. This suggests that the static magnetic field with ultrasonic pretreatment assistance had various effects on the TA. Table 1 shows that, following freezing and thawing, CF had the lowest ascorbic acid level, which was significantly (*p* < 0.05) lower than MF, U-CF, and U-MF. The ascorbic acid content of MF, U-CF, and U-MF showed no significant difference (*p* > 0.05). The ascorbic acid content of frozen–thawed blueberries declined compared with fresh blueberries, which could be because ascorbic acid is water soluble and can be very easily lost with the loss of juice from frozen–thawed samples [26]. This observation is consistent with the findings of a previous study on frozen honeydew melon [5]. In addition, relevant studies have indicated that the enzymatic activity was enhanced in the thawed samples, and that the enzymatic and nonenzymatic physicochemical reactions were accelerated, resulting in the decomposition of ascorbic acid, which is another pathway for the loss of ascorbic acid [27]. It can be found that the MF, U-CF, and U-MF treatments significantly increased the ascorbic acid content of the samples compared with CF. This indicates that the ultrasound pretreatment-assisted static magnetic field was effective in increasing the ascorbic acid content of the blueberry samples. In addition, Ref. [28] indicated that a static magnetic field significantly increased the ascorbic acid content of thawed broccoli and cauliflower. According to Table 1, no significant differences were observed in the reducing sugar contents of blueberries treated with CF and MF (*p* > 0.05), or those of blueberries treated with U-CF and U-MF. The obtained parameters showed that the static magnetic field had no significant effect on the reducing sugar content. However, ultrasound treatment significantly increased the reducing sugar content. The reducing sugar content was increased by 8.44% in U-CF compared with CF, and increased by 4.94% in U-MF compared with MF. This might be because the ultrasound treatment further accelerated the penetration of trehalose, resulting in an increase in reducing sugar content. A previous study reported that ultrasound at a frequency of 20 kHz and a power of 500 W was used to treat cassava starch, leading to an enhancement in the reducing sugar content [29].

After freezing, the reducing sugar content increased and the organic acid content decreased in blueberry fruits, which improved of fruit sugar-to-acid ratio of the blueberries. The ascorbic acid content was significantly reduced due to the loss of juice. Overall, U-MF better maintained the TSS, ascorbic acid, and reduced sugar contents in thawed blueberries.

### 3.4. Organic Acid Content

**Figure 2:**
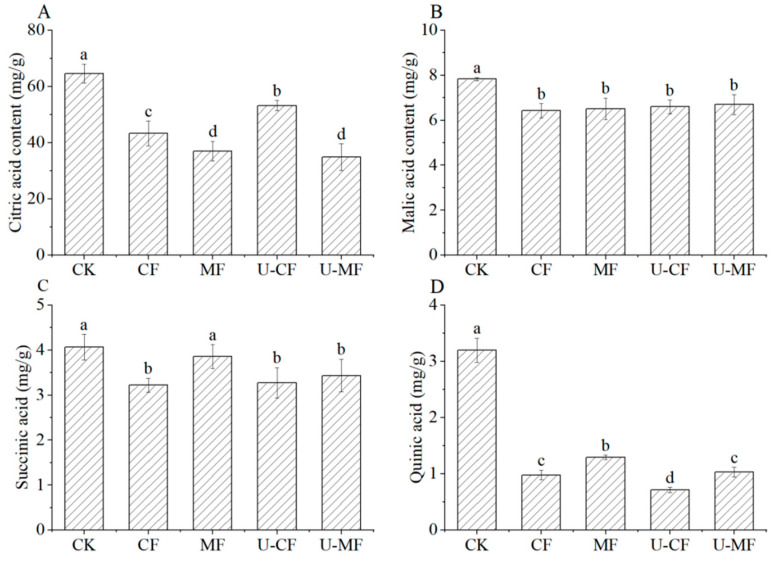
Organic acid contents of blueberries in different treatment groups. CK: fresh blueberries; CF: control freezing (−20 °C, 0 mT); MF: static magnetic field freezing (−20 °C, 10 mT); U-CF: ultrasound pretreatment + control freezing; U-MF: ultrasound pretreatment + static magnetic field freezing. All frozen groups were stored at −18 ± 1 °C for 72 h before analysis. (**A**) Citric acid; (**B**) malic acid; (**C**) succinic acid; (**D**) quinic acid. Different letters indicate significant differences (*p* < 0.05).

One important factor affecting the nutritional content and flavor of fruits is their organic acid composition [30]. Organic acids are key intermediates in the tricarboxylic acid (TCA) cycle, which regulates energy metabolism in blueberry cells. Their retention reflects the maintenance of cellular metabolic integrity during freezing. Furthermore, organic acids enhance the order and stability of anthocyanins through hydrogen bonding and π-π stacking formed by molecular docking, thereby reducing their degradation into colorless forms. Fruits rich in organic acids can enhance the desire for food, help digestion in the gastrointestinal tract, and improve the stability of vitamins and other substances, which is conducive to better absorption and utilization in the human body. Figure 2 demonstrates the change in the citric, malic, succinic, and quininic acid contents in blueberries. It can be seen from Figure 2 that the highest organic acid was citric acid in the ‘Hokuriku’ blueberry, followed by malic, succinic, and quininic acids. Ref. [31] reported that the most abundant organic acid in ‘Powderblue’ blueberry was malic acid, followed by quininic, citric, and succinic acids. The contents and types of organic acids were affected by the variety, genotype, and growth environment in blueberries. The organic acid contents of fresh blueberries were higher compared with other freezing blueberries. This might be due to the loss of fruit juice after freezing, as well as the internal nutrients of the fruits, such as organic acids, which are also lost with the loss of water, thereby leading to a reduction in the organic acid content of frozen blueberries. Ref. [32] also observed a reduction in total organic acids in the “Chester Thornless” and “Thornless Evergreen” blackberry fruits due to −20 °C freezing and long-term frozen storage. As shown in Figure 2A, after freezing and thawing, the content of citric acid declined significantly compared with fresh blueberries. The citric acid content of U-CF was relatively higher than that of MF and U-MF (*p* < 0.05). According to Figure 2B, the malic acid content had no significant effect (*p* > 0.05) in all frozen blueberries. Figure 2C shows that succinic acid demonstrated a significant decrease in CF, U-CF and U-MF frozen groups compared with fresh blueberries (*p* < 0.05). There was no significant difference (*p* > 0.05) in the succinic acid of CF and U-CF, and U-MF, but there was a significant difference (*p* < 0.05) with MF. Compared with CF, the content of quininic acid in MF increased, while it was reduced in U-CF (Figure 2D) and showed no significant difference with U-MF. This suggested that the static magnetic field maintained the quinic acid content, and that the ultrasound pretreatment accelerated the decomposition of quinic acid. Some studies reported that fruit senescence and storage characteristics are affected by organic acids [33,34]. Moreover, citric and malic acids are intermediates of plant cellular respiratory metabolism and play a crucial role in the material and energy metabolism in plants. Citric and malic acids transform into each other, and juice loss leads to a reduction in the citric and quininic acid contents after freezing. Ultrasonic pretreatment disrupts cellular structures through cavitation effects, releasing organic acids while reducing their exposure to degradative enzymes. Concurrently, a static magnetic field delays acid oxidation by regulating the ice crystal morphology, inhibiting free radical generation, and enhancing antioxidant enzyme activity. This synergistic approach optimizes ice nucleation and cellular permeability, thereby effectively retarding the degradation of citric, succinic, and quinic acids.

### 3.5. Antioxidant Capacities

**Figure 3:**
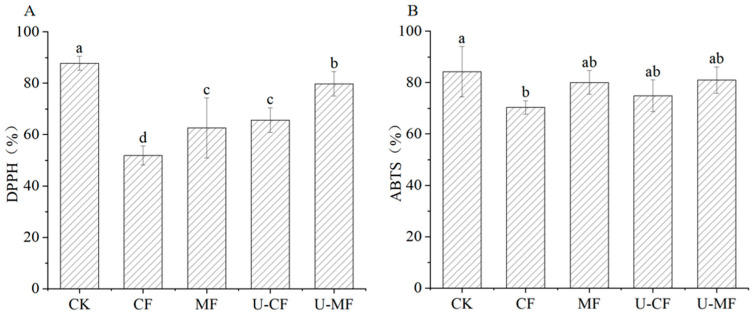
DPPH (**A**) and ABTS (**B**) radical scavenging capacities of blueberries in different treatment groups. CK: fresh blueberries; CF: control freezing (−20 °C, 0 mT); MF: static magnetic field freezing (−20 °C, 10 mT); U-CF: ultrasound pretreatment + control freezing; U-MF: ultrasound pretreatment + static magnetic field freezing. All frozen groups were stored at −18 ± 1 °C for 72 h before analysis. Different letters indicate significant differences (*p* < 0.05).

DPPH and ABTS are nitrogen-centered, lipid-soluble free, and water-soluble radicals, which are frequently employed to assess the short-term antioxidant capability of samples [35]. Figure 3 shows the changes in the DPPH and ABTS radical scavenging capacities of fresh and frozen blueberries. According to Figure 3, the DPPH values of the frozen treatments demonstrated a decline after freezing and thawing. The DPPH value of frozen blueberries treated with a static magnetic field and the ultrasound pretreatment increased significantly compared with CF. The DPPH value of U-MF was the highest, followed by U-CF, and MF, while CF was the lowest in the frozen groups. The DPPH value of MF, U-CF, and U-MF showed a significant difference (*p* < 0.05) compared with CF. This indicates that the ultrasound pretreatment-assisted static magnetic field had a significant effect on the DPPH activity. Compared with CF, there were no significant variations in the ABTS values of blueberries in the MF, U-CF, and U-MF groups (*p* > 0.05; Figure 3), indicating that the ultrasound pretreatment in combination with static magnetic field-assisted freezing had no significant effect on the ABTS activity. Ref. [7] found that ultrasound (21 kHz and 30 min) increased the DPPH activity of apples, but had no significant effect on the ABTS activity.

### 3.6. Color

The flesh color of fruits is a crucial characterization to assess fruit quality and determine the quality of processed foods and raw materials [36]. Table 2 elucidates the change in flesh color attributes in fresh and frozen blueberries. It can be seen from Table 2 that the flesh color of freeze–thawed blueberries changed significantly (*p* < 0.05) compared with fresh ones, which led to a darker color. After freezing and thawing, the L* value of the blueberry flesh decreased remarkably in comparison to the fresh blueberry (*p* < 0.05), indicating that the brightness of the blueberry samples decreased. This could be due to enzymatic browning reactions catalyzed by polyphenol oxidase and peroxidase [36], producing dull and lusterless blueberry flesh. MF, U-CF, and U-MF were characterized by lower L* values than those of CF. The obtained parameters indicated that ultrasound pretreatment-assisted static magnetic field freezing reduced the L* value of blueberry flesh. In a previous study conducted by [37], the L* value of apples processed using a magnetic field decreased significantly due to the inhibitory effect on polyphenol oxidase and peroxidase activities. The a* value was changed from green to red. The U-CF and U-MF exhibited a higher degree of transformation after freezing. The a* of blueberry flesh changed from a negative to a positive value. The alteration of the a* value may be involved in the production of colored compounds because of non-enzymatic and enzymatic browning during processing [5]. Furthermore, Ref. [7] treated apples with contact ultrasound and found a decrease in the L* value and an increase in the a* value compared to the control group. Table 2 also displays that the b* value of the flesh color was reduced. The reason for this is that freezing and thawing destroy the complete structure of blueberry cells. The reduction in the b* value after thawing could be due to relatively higher degree of browning of the blueberry flesh, which leads to the weakening of the yellow color.

The ΔE* value of other frozen blueberries increased significantly (*p* < 0.05) compared with CF, indicating that a static magnetic field and ultrasound can considerably alter the flesh color of fruits. This is because it is easy for enzymes to come into contact with the browning substrates in blueberries after freezing and thawing, which leads to the occurrence of enzymatic browning [38]. In addition, freezing exacerbates the destruction and breakdown of pigments, and water-soluble pigments are lost with juice loss [39].

### 3.7. Analysis of Correlation

**Figure 4:**
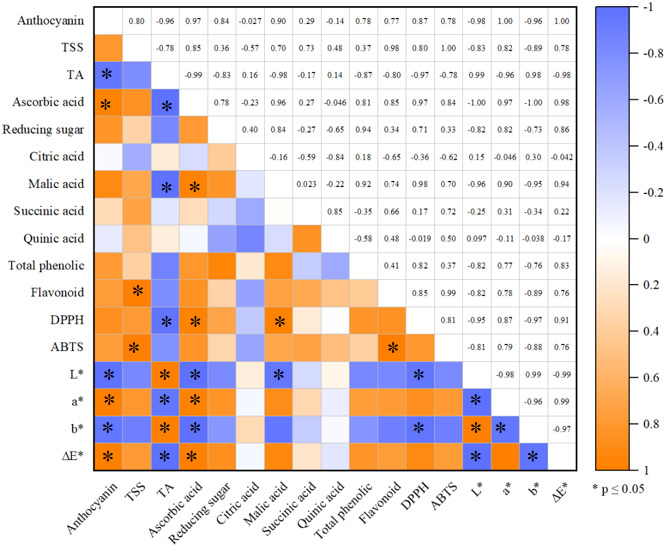
Pearson correlation analysis of various indicators. CK: fresh blueberries; CF: control freezing (−20 °C, 0 mT); MF: static magnetic field freezing (−20 °C, 10 mT); U-CF: ultrasound pretreatment + control freezing; U-MF: ultrasound pretreatment + static magnetic field freezing. All frozen groups were stored at −18 ± 1 °C for 72 h before analysis. Each square with a different color indicates the correlation coefficient. The orange (+1) and blue (−1) colors represent positive and negative correlations. Asterisks (*) represent significant differences (*p* < 0.05).

Variations in the contents of phenolic compounds in blueberries after freezing and thawing, as well as the antioxidant capacity, were compared using a heatmap. Correlation coefficients between the indicators are shown in Figure 4. From the results in Figure 4, the correlation analysis suggested that the anthocyanin content exhibited a positive correlation with ascorbic acid, a*, and ΔE* in blueberries, suggesting that the anthocyanin content in blueberry fruit was strongly and positively correlated with color during freezing. The TA content was positively correlated with L* and b* values (r = 0.99 and 0.98, respectively). It was observed that the coefficient ΔE*, a*, DPPH, malic acid, and ascorbic acid showed a significantly positive correlation, suggesting that the ascorbic acid content of blueberry fruit was significantly and positively associated with the antioxidant capacity and color during freezing. In addition, the flavonoid content and ABTS also showed a strong positive correlation. The L* value of blueberries was positively correlated with the b* value, and the a* value was positively correlated with the ΔE* value (r = 0.99). In conclusion, freezing has a great effect on the bioactivity, antioxidant capacity, and flesh color of blueberries during freezing and thawing. Ultrasound-assisted freezing treatment can maintain higher levels of bioactivity and accelerate flesh color change in blueberries.

## 4. Conclusions

This study explored the effect of ultrasound pretreatment and a static magnetic field freezing on the bioactive compounds, antioxidant properties, organic acids, and flesh color of blueberries. The results of this study demonstrated that ultrasonic pretreatment combined with static magnetic field freezing technology significantly preserved higher levels of key bioactive compounds compared to the control freezing group (CF, −20 °C). The anthocyanin content increased by 33.67%, the total phenolic content rose by 29.14%, and the flavonoid content grew by 18.65% (*p* < 0.05). Ultrasonic pretreatment disrupted blueberry cell walls, enhancing the extractability of bound phenolic compounds. Concurrently, the static magnetic field reduced the ice crystal size by altering hydrogen bonding in the water molecules. This minimized cellular damage and decreased the leaching of water-soluble compounds—such as anthocyanins and ascorbic acid—during thawing. Ultrasound pretreatment combined with static magnetic field-assisted freezing had a significant effect on the DPPH antioxidant activity of frozen blueberries, but had no effect on the ABTS antioxidant activity of frozen blueberries. To summarize, ultrasound pretreatment-assisted static magnetic field freezing had a positive effect on the physicochemical characteristics of the blueberries, synergistically preserving the bioactive compounds, antioxidant capacity, and flavor quality. The findings of the present study provide a theoretical basis for a promising freezing technology for preserving fruits and vegetables.

## Figures and Tables

**Table 1:** Effects of different treatment groups on the TSS, TA, ascorbic acid, and reducing sugar content levels of blueberries. CK: fresh blueberries; CF: control freezing (−20 °C, 0 mT); MF: static magnetic field freezing (−20 °C, 10 mT); U-CF: ultrasound pretreatment + control freezing; U-MF: ultrasound pretreatment + static magnetic field freezing. All frozen groups were stored at −18 ± 1 °C for 72 h before analysis.

Sample	TSS (%)	TA (%)	Ascorbic Acid (mg 100g^−1^)	Reducing Sugar Content (%)
CK	10.91 ± 0.29 ^c^	0.66 ± 0.05 ^a^	22.44 ± 1.56 ^a^	5.68 ± 0.26 ^c^
CF	11.31 ± 0.31 ^b^	0.31 ± 0.05 ^b^	6.29 ± 0.87 ^c^	8.18 ± 0.34 ^b^
MF	11.81 ± 0.23 ^a^	0.21 ± 0.02 ^c^	7.57 ± 0.86 ^b^	8.30 ± 0.48 ^b^
U-CF	11.57 ± 0.37 ^a,b^	0.16 ± 0.05 ^d^	7.88 ± 0.94 ^b^	8.87 ± 0.48 ^a^
U-MF	11.83 ± 0.35 ^a^	0.10 ± 0.02 ^e^	8.58 ± 1.47 ^b^	8.71 ± 0.32 ^a^

Data represent the mean ± standard deviation from three independent replicate determinations; distinct letters within the same column denote statistically significant differences (*p* < 0.05).

**Table 2:** Influence of different treatment groups on the flesh color in blueberries. CK: fresh blueberries; CF: control freezing (−20 °C, 0 mT); MF: static magnetic field freezing (−20 °C, 10 mT); U-CF: ultrasound pretreatment + control freezing; U-MF: ultrasound pretreatment + static magnetic field freezing. All frozen groups were stored at −18 ± 1 °C for 72 h before analysis.

Sample	L*	a*	b*	ΔE*
CK	46.35 ± 1.76 ^a^	−4.15 ± 0.96	16.19 ± 1.25 ^a^	51.29 ± 1.31 ^d^
CF	33.69 ± 1.51 ^b^	5.24 ± 0.99 ^c^	10.21 ± 1.17 ^b^	61.78 ± 1.41 ^c^
MF	31.35 ± 1.37 ^c^	8.31 ± 0.98 ^b^	8.26 ± 1.08 ^c^	64.08 ± 1.33 ^b^
U-CF	30.59 ± 0.99 ^d^	9.22 ± 0.71 ^a^	8.10 ± 0.56 ^c^	65.14 ± 1.30 ^a^
U-MF	29.65 ± 0.62 ^e^	9.52 ± 0.81 ^a^	6.95 ± 0.50 ^d^	65.58 ± 0.79 ^a^

Values are expressed as mean values ± standard deviation, with three replicates performed for each measurement; different superscript letters in the same column signify significant differences at the *p* < 0.05 level.

## Data Availability

The original contributions presented in this study are included in the article; further inquiries can be directed to the corresponding author.
